# How Do Accountable Care Organizations Deliver Preventive Care Services? A Mixed-Methods Study

**DOI:** 10.1007/s11606-019-05271-5

**Published:** 2019-08-20

**Authors:** Adam D. M. Briggs, Taressa K. Fraze, Andrew L. Glick, Laura B. Beidler, Stephen M. Shortell, Elliott S. Fisher

**Affiliations:** 1 The Dartmouth Institute for Health Policy and Clinical Practice, Dartmouth College, Lebanon, NH USA; 2 The Nuffield Department of Population Health, University of Oxford, Old Road Campus, Headington, Oxford, OX3 7LF UK; 3 University of Connecticut School of Medicine, Farmington, CT USA; 4 School of Public Health, University of California, Berkeley, CA USA

**Keywords:** prevention, accountable care organizations, Medicare, preventive care, health care reform

## Abstract

**Background:**

The Affordable Care Act and the introduction of accountable care organizations (ACOs) have increased the incentives for patients and providers to engage in preventive care, for example, through quality metrics linked to disease prevention. However, little is known about how ACOs deliver preventive care services.

**Objective:**

To understand how Medicare ACOs provide preventive care services to their attributed patients.

**Design:**

Mixed-methods study using survey data reporting Medicare ACO capabilities in patient care management and interviews with high-performing ACOs.

**Participants:**

ACO executives completed survey data on 283 Medicare ACOs. These data were supplemented with 39 interviews conducted across 18 Medicare ACOs with executive-level leaders and associated clinical and managerial staff.

**Main Measures:**

Survey measures included ACO performance, organizational characteristics, collaboration experience, and capabilities in care management and quality improvement. Telephone interviews followed a semi-structured interview guide and explored the mechanisms used, and motivations of, ACOs to deliver preventive care services.

**Key Results:**

Medicare ACOs that reported being comprehensively engaged in the planning and management of patient care - including conducting reminders for preventive care services - had more beneficiaries and had a history of collaboration experience, but were not more likely to receive shared savings or achieve high-quality scores compared to other surveyed ACOs. Interviews revealed that offering annual wellness visits and having a system-wide approach to closing preventive care gaps are key mechanisms used by high-performing ACOs to address patients’ preventive care needs. Few programs or initiatives were identified that specifically target clinically complex patients. Aside from meeting patient needs, motivations for ACOs included increasing patient attribution and meeting performance targets.

**Conclusions:**

ACOs are increasingly motivated to deliver preventive care services. Understanding the mechanisms and motivations used by high-performing ACOs may help both providers and payers to increase the use of preventive care.

## INTRODUCTION

The Affordable Care Act (ACA) has led to several key policy changes that incentivize both patients and providers to engage more in preventive care. For patients, the ACA removed cost sharing for a wide range of primary and secondary disease prevention services, and it introduced Medicare annual wellness visits (AWVs) that focus on prevention.^1, 2^ For providers, Medicare began to offer substantial reimbursement for AWVs and link pay to preventive care quality measures.^2–4^ But despite these changes, uptake of preventive care services has been slow and few patients take up their full entitlement of disease prevention interventions.^5–10^

In 2018, accountable care organizations (ACOs) provided health care for over 10% of the US population, with coverage growing steadily since they were first introduced in 2011.^11^ ACOs may help improve the uptake of preventive care services among their attributed patients because health care providers working within ACO contracts have greater incentives than under traditional fee-for-service to prevent disease and improve health. Firstly, specific preventive care services are commonly included among the quality metrics for which ACOs are held accountable, and secondly, screening and immunizations may save the ACO money through reduced expenditure on preventable conditions and complications.^4, 12^ But despite these incentives, how ACOs meet the preventive care needs of their patients remains largely unknown. One study found that Medicare ACOs with high preventive care quality scores had more beneficiaries per full-time equivalent (FTE) primary care provider,^13^ and another showed that Medicare ACO patients are 50% more likely to have an AWV than non-Medicare ACO patients.^14, 15^

In this study, we aim to understand how Medicare ACOs provide preventive care services. We focus on clinical preventive services aimed at primary and secondary disease prevention (for example, vaccinations and screening) and targeting harm reduction from unhealthy behaviors. We used a mixed-methods approach. Using survey data, we compared the characteristics of ACOs that do and do not report having a comprehensive approach to care management and disease prevention. We then conducted interviews with a subset of survey respondents to understand how ACOs deliver preventive care services to their attributed patients.

## METHODS

We used both quantitative and qualitative data. Survey data were used to characterize differences between Medicare ACOs that report taking a comprehensive approach to patient care planning and management, including addressing patients’ preventive care needs, and Medicare ACOs that do not. These data provided context for understanding and interpreting telephone interviews with ACO leaders and other associated clinical and managerial staff from a subset of survey respondents. Interviewees were asked about how they manage the care of their patients, including the delivery of preventive services, and focused on patients with complex needs.

### Survey Data

The National Survey of ACOs (NSACO) was used to describe the organizational characteristics, payment experience, performance, and capabilities of Medicare ACOs taking a comprehensive approach to patient care management, including preventive care.

The NSACO is a web-based survey of all newly formed ACOs (both Medicare and non-Medicare) starting in August 2012. We used data on Medicare ACOs, including Medicare Shared Savings Program participants and Medicare Pioneer ACOs, from the first three waves of the NSACO (October 2012 to May 2013, September 2013 to March 2014, and November 2014 to April 2015); each ACO was only invited to participate in one of the three waves. An executive-level leader (for example, an ACO director or Chief Medical Officer) was invited to participate and the total response rate to the three waves was 64%. A detailed description of the survey and its data has been published previously,^16^ and no evidence of non-response bias has been identified for key variables for each of the three waves.^16–18^ Non-response bias for each wave was tested by comparing the sample distribution of key variables—for example, beneficiary composition, savings, and quality performance—among ACOs who responded to the survey with all ACOs; no differences were observed.

Medicare Centers for Medicaid and Medicare Services (CMS) data from 2015 on ACO size, performance, and financial management was matched to ACOs surveyed by NSACO to identify which surveyed ACOs achieved shared savings and ACOs’ mean overall quality score for those reporting in that year. Table 1 shows the baseline characteristics of Medicare ACOs that responded to the survey. Survey data were analyzed using Stata, release 15,^19^ with response differences assessed using a chi-squared test unless otherwise specified.

**Table 1:** Characteristics of Surveyed and Interviewed Medicare ACOs

	Medicare NSACO respondents	Outreach sample for interview	Interview sample
Payer Mix	*n* = 297	*n* = 50	*n* = 18
Any private payer contract	48%	42%	67%
Any Medicaid contract	18% (*n* = 289)	20%	33%
Only Medicare contract	44%	54%	28%
Contract with > 1 payer	56%	46%	72%
Composition	*n* = 263	*n* = 50	*n* = 18
Mean (SD) number of PCPs	139 (153)	187 (197)	189 (157)
Mean (SD) number of specialists	294 (472)	353 (447)	388 (508)
Mean (SD) number of attributed beneficiaries	16,237 (14,438)	21,003 (22,153)	19,185 (15,126)
Beneficiary:PCP ratio (SD)	167 (105)	113 (115)	142 (85)
Mean (SD) number of facilities	5.0 (15)	5.4 (18)	6.6 (12)
Number with a hospital	57% *(n = 278)*	42%	56%
Region	*n* = 283	*n* = 50	*n* = 18
Northeast	24%	16%	33%
South	37%	50%	33%
Midwest	23%	18%	22%
West	15%	16%	11%
Performance—Medicare			
Achieved savings year 1	25% (*n* = 234)	58% (*n* = 45)	53% (*n* = 15)
Achieved savings year 2	31% (*n* = 225)	58% (*n* = 45)	60% (*n* = 15)
Achieved savings year 3	40% (*n* = 151)	78% (*n* = 32)	70% (*n* = 10)
Mean (SD) 2015 quality score	92 (9) (*n* = 207)	93 (6) (*n* = 44)	95 (3) (*n* = 15)

The number of ACOs included in each row varies depending on data availability

*NSACO* National Survey of Accountable Care Organizations, *PCP* primary care provider, *SD* standard deviation

Of the 297 Medicare ACOs that responded, 283 answered the question: “To what extent are providers engaged in planned and continuous management of patient care?” Medicare ACOs that responded that “comprehensive pre–visit planning, medication management and review, and reminders for preventive care and specific tests are conducted” were compared with those that reported not using a comprehensive approach. As with previous analyses of the NSACO, we describe how each group differs in terms of composition, payment reform and collaboration experience, performance, clinician management, care management capabilities, approach to quality improvement, patient engagement, and use of health information technology.^20^ We also analyzed 2015 overall and preventive care quality scores (an average of the eight preventive care quality scores: breast and colorectal cancer screening, influenza and pneumococcus vaccination, and BMI, tobacco, depression, and blood pressure screening^13, 21^) and success at achieving shared savings in 2015. The measures were chosen because they were hypothesized to be associated with patient care management and preventive care quality.^13^ Of note, 29 of the 33 CMS quality measures used in 2015 are unadjusted for patient case-mix, exceptions are the two measures of readmissions and the two ambulatory sensitive conditions admission measures.^21^

### Interview Data

To complement the quantitative data, between February 2018 and June 2018 we conducted 39 semi-structured telephone interviews with ACO leaders and with clinical or managerial staff based at either the ACO or an ACO member organization. Interviewees were executive-level leaders at 18 ACOs. Eleven of the 18 ACOs agreed to additional interviews with clinical or managerial staff in order to gain detail about specific care programs and services. The fifty Medicare NSACO respondents that had achieved shared savings at least once were invited to participate and sixteen agreed (see Table 1 for characteristics of invited Medicare ACOs and those who participated). One further ACO contacted us independently to participate, and another agreed to interview following additional invitations sent to ACOs with high CMS preventive care quality scores. All had achieved shared savings.

Interviews lasted for approximately 1 hour and included questions on the ACO’s composition, leadership and partnerships, the care of complex patients, relationships between ACOs and participating practices, and future plans. Interviewees were asked if their ACO has “any programs or initiatives aimed at providing care or preventing disease for (1) patients with complex chronic conditions, (2) the frail elderly, (3) patients with mental or behavioral illness, (4) hospital high utilizers, (5) any other patients you consider complex.” This paper reports on responses that describe clinical activities related to the primary or secondary prevention of disease, or that aim to reduce the impact of unhealthy behaviors.

Table 1 shows ACO characteristics of those interviewed compared with the NSACO sample. The study protocol was approved by Dartmouth College’s Institutional Review Board.

All interviews were recorded and transcribed. Transcripts were analyzed using QSR NVivo software.^22^ Coding for broad topic areas (such as preventive care) was conducted by three team members; after reviewing three transcripts and reconciling differences in broad topic coding practice, each transcript was coded by two team members.^23^ Detailed coding of preventive care themes was done using an iterative process whereby two team members initially coded all data related to preventive care into enablers and barriers, and then again for proposed themes as they emerged. Coding discrepancies were identified and discussed to achieve consensus.

## RESULTS

### Survey Results

Of the 283 Medicare ACOs responding to the NSACO question about how their providers engage in the planning and continuous management of patient care, 25% reported having “comprehensive pre-visit planning, medication management and review, and conduct reminders for preventive care and specific tests.” These ACOs were more likely to have more primary care physicians (PCPs), be physician-led, and have more beneficiaries than ACOs less engaged in planning and continuous patient management (although there was no difference in beneficiary:PCP ratio; Tables 2 and 3). Survey results suggest that although these ACOs report higher capabilities in various aspects of care management, performance management, quality improvement, and patient activation and engagement, there was no association with higher overall or preventive care quality scores, or a higher likelihood of achieving shared savings. There was also no difference between the ACO groups analyzed in terms of their participation in different payment reforms, approach to physician performance and compensation, and use of health information technology.

**Table 2:** Characteristics and Quality and Savings Performance of Medicare NSACO Respondents

	Is comprehensively engaged in patient care planning and continuous management (*n* = 70)	Is not comprehensively engaged in patient care planning and continuous management (*n* = 213)	*P* value of difference (chi^2^ unless specified)
ACO contracts			
Medicare only	46%	44%	0.764
Has Medicaid contract	17%	18%	0.915
Has commercial contract	49%	50%	0.862
Medicare two-sided risk	6%	2%	0.095
Composition			
Mean (SD) number of FTE PCPs	216 (289)	146 (158)	0.013*
Mean (SD) number of FTE specialists	292 (407)	227 (376)	0.267*
Mean (SD) 2015 attributed Medicare beneficiaries	25,007 (29,755)	18,665 (16,762)	0.043*
Beneficiary:PCP ratio (SD)	181 (233)	221 (420)	0.515*
Physician-led	77%	53%	< 0.001
Hospital-led	0%	7%	0.028
Has a hospital	54%	59%	0.503
Part of integrated delivery system	47%	48%	0.858
Percentage of primary care patient panel covered by ACO contracts			
0–24%	47%	51%	0.396
25–49%	34%	39%	
50–74%	13%	6%	
75–100%	6%	4%	
Medicare performance			
Achieved shared savings in 2015	23%	33%	0.167
Mean (SD) 2015 quality score	91 (10)	92 (9)	0.401*
Mean (SD) 2015 CMS quality score among preventive care domains^13^	65 (11)	67 (11)	0.491*

*NSACO* National Survey of Accountable Care Organizations, *CMS* Center for Medicare and Medicaid Services, *FTE* full-time equivalent, *PCP* primary care practitioner, *SD* standard deviation

*Denotes use of two-sided *t* test

**Table 3:** Experience and Capabilities of Medicare NSACO Respondents

	Is comprehensively engaged in patient care planning and continuous management (*n* = 70)	Is not comprehensively engaged in patient care planning and continuous management (*n* = 213)	*P* value of difference (chi^2^ test)
Payment reform and collaboration experience			
Bundled or episode-based payments experience	34%	34%	0.957
Patient-centered medical home experience	89%	83%	0.281
Pay for performance experience	94%	86%	0.105
Public reporting experience	89%	84%	0.429
Capitated commercial contracts experience	46%	47%	0.902
Other risk-bearing contracts experience	60%	57%	0.686
Previous close collaboration between participating organizations	46%	25%	0.004
Advanced capabilities in quality performance measurement and financial rewards			
Actively monitors performance and provides clinician feedback	71%	42%	< 0.001
Comprehensive and timely financial performance	43%	24%	0.002
Physician quality performance reported and shared	78%	71%	0.250
Physician cost performance reported and shared	46%	53%	0.349
One-on-one physician review and feedback	62%	63%	0.921
Individual physician financial incentives	41%	40%	0.977
Individual non-financial awards	25%	20%	0.383
Advanced capabilities in care management			
Chronic care management process and programs	71%	23%	< 0.001
Smooth transitions of care	50%	15%	< 0.001
Assessing provider quality and cost	40%	10%	< 0.001
Integrated behavioral health programs	31%	6%	< 0.001
Patient involvement in care decisions	48%	14%	< 0.001
Established planning of end-of-life care	41%	12%	< 0.001
Advanced capabilities in quality improvement			
Use choosing wisely	35%	25%	0.144
Use evidence-based guidelines	97%	98%	0.793
Program to reduce preventable hospital readmissions	59%	39%	0.003
Standardized processes and guidelines	44%	13%	< 0.001
Engaged in programs to reduce ambulatory care sensitive admissions	63%	32%	< 0.001
Assess inappropriate ED use	60%	38%	0.002
Using disease monitoring data	76%	46%	< 0.001
Measuring patient satisfaction	67%	44%	0.002
Clinician training in QI methods	48%	21%	< 0.001
Communication across care settings	43%	20%	0.005
Advanced capabilities in patient activation and engagement			
Most PCPs trained in patient activation and engagement methods	69%	64%	0.517
Most PCPs work with patients to develop treatment plan	79%	71%	0.238
Most high-risk patients engage in care transition program	80%	72%	0.201
Most PCPs offer patients evidence-based decision aids	80%	67%	0.041
Offer all clinicians training in shared decision-making	22%	4%	0.001
Advanced capabilities in health information technology			
Use of single EHR	20%	22%	0.772
Meaningful use of EHR by majority of PCPs	59%	55%	0.681
Can run predictive risk assessment and stratification	47%	35%	0.093

*ED* emergency department, *EHR* electronic health record, *NSACO* National Survey of Accountable Care Organizations, *PCP* primary care practitioner, *QI* quality improvement

### Interview Findings

Although our quantitative results failed to find an association between reporting comprehensive approaches to care management and higher CMS-reported overall or preventive care quality, our qualitative data show that for those ACOs interviewed, care management was a key mechanism for delivering preventive care services and that performance on quality indicators was an important motivator for their efforts.

Of the 18 Medicare ACOs interviewed, 15 provided information on how they deliver preventive care services. The two key mechanisms used were the Medicare annual wellness visit and closing preventive care gaps, for example, missed screening tests or vaccinations. Interviews provide insights into ACOs’ motivations for providing preventive care services, and two key facilitating factors that help to support preventive care delivery are also identified. Table 4 summarizes the principal interview findings using this framework.

**Table 4:** Preventive Care Activities Used by ACOs, Their Motivations, and Facilitating Factors

	Annual wellness visits	Closing preventive care gaps
Mechanisms	• Delivering the Medicare annual wellness visit	• Closing care gaps among targeted patient groups
		• Closing care gaps through care management and care coordination programs
		• Closing care gaps as part of routine clinical care
Motivations	• Doing what is best for the patient	• Doing what is best for the patient
	• Meeting targets	• Meeting targets
	• Achieving patient attribution	
	• Coding disease complexity	
	• Reimbursement opportunity	
Facilitators	• ACO and practice education and training	
	• Use of technology and the electronic health record	

#### Annual Wellness Visits

Nine ACOs highlighted the use of AWVs to provide preventive care services. The AWV is a yearly checkup offered by Medicare—with no patient co-pay—to screen for modifiable health risks and provide preventive disease services. It is separate and in addition to an annual physical examination.^24^ We identified five motivations of ACOs to conduct AWVs, listed in Table 4.

ACOs were in part motivated by doing what they felt was best for their patient. For example, one ACO leader told us that the AWV is a valuable opportunity to identify their patients’ overall care needs for the coming year, to have a conversation “where it’s not just a sick visit.”

As well as addressing patients’ preventive care needs, interviews highlighted four other motivations for delivering AWVs. Firstly, the AWV is used to fill care gaps for preventive care services included within CMS quality outcomes measures, thereby improving quality scores and the proportion of shared savings earned.^4^ Secondly, AWV billing codes are recognized as a primary care service by CMS when assigning beneficiaries to a Medicare ACO making the AWV a vehicle for increasing or maintaining patient attribution.^25^ An ACO care coordination manager summarized both of these motivations:

And so you don't want your attributions to drop and by getting [patients] in for their annual wellness visits, that helps keep them attributed to the practice as well as it gives you that opportunity to address any of the quality measures that CMS is gonna be looking at when they audit to see if, okay, maybe [patient x] is due for a mammogram, well this is the time that we can address those and let’s get her set up for that.

Thirdly, AWVs present an opportunity for accurately coding patients’ clinical conditions (using Hierarchical Condition Category (HCC) risk scores) that are then used to risk-adjust the ACO’s benchmark against which CMS calculates savings or losses at the end each year.^25^ As one ACO director told us:

[Completing the AWV before the physical] means that everything is nicely packaged up, so we’ve identified gaps in care, we’ve tried to close them, we’ve looked at the RAF [risk adjustment factor] scoring, we’ve done the medication reconciliation, we have provided them with a personalized care plan, their immunization schedule is done.

Finally, CMS offers a relatively high reimbursement rate for AWVs compared to most other primary care services.^15^ One ACO leader recognized how important this is to clinicians, describing AWVs as “RVU-rich.” This ACO worked to ensure that the electronic health record (EHR) could capture all the information required to complete an AWV and be reimbursed by CMS. A second ACO leader described how their ACO had centralized the organization and delivery of AWVs, removing the responsibility (and billing capability) away from individual practices. This was done to improve the consistency and quality of visits, ensuring that all CMS requirements for billing and performance were met. However, before the program could be fully implemented across all their patients, the ACO leadership had to agree to cover practices’ lost potential revenue.

#### Closing Preventive Care Gaps

The second key mechanism used by ACOs is closing preventive care gaps, for example, identifying and addressing missed screening tests or vaccinations, mentioned by 10 of the ACOs interviewed. Responses can be further divided into three groups of activities: activities that target specific patient groups who are known to, or likely to, have gaps in their uptake of preventive care services; closing preventive care gaps as part of care management and care coordination programs; and closing preventive care gaps as part of routine clinical care.

##### Closing Preventive Care Gaps Among Targeted Patient Groups

Several ACOs discussed how they would actively identify patients with outstanding preventive care needs or would link preventive care services to significant life events or national campaigns. Other ACO leaders discussed how their data systems can be used to generate lists of specific patients with preventive care gaps. These patients are then followed up either by a quality team within the ACO or by the practice.

##### Closing Preventive Care Gaps Through Care Management and Care Coordination Programs

Care management and care coordination programs typically facilitate or provide support for patients with ongoing or complex clinical needs. The following quote from an ACO leader is a typical example:

[The care coordinator] may be calling just to say, ‘You know what, you’re overdue for your diabetes visit or [your physician] wanted to see you in six weeks instead of three months because you’re A1C was elevated.’ They’re doing a lot of that. They’ll do some behavioral activation…They may call up and say, ‘Hey, how are you doing with this diet or this exercise program?’

##### Closing Preventive Care Gaps as Part of Routine Clinical Care

ACOs also described how preventive care gaps are closed as part of routine clinical care. This was often helped by medical assistants or computer systems prompting clinical staff when gaps exist. For example, one physician told us about their medical assistant who sits in on all routine patient encounters both to act as a scribe and to remind the physician about what payment contract the patient is under and to close relevant care gaps.

The importance of taking a system-level approach to closing preventive care gaps in all aspects of clinical work was emphasized by another ACO leader who told us how their physicians’ pay is directly linked to closing care gaps.

##### Motivations

The motivations for closing preventive care gaps were again a desire to do what is best for patients and to meet performance targets.

#### Factors Facilitating Preventive Care Delivery

Interviews identified two facilitating factors that, if available, can support the delivery of preventive care services. The first is the use of education and training and the second is the use of technology.

##### ACO and Practice Education and Training

The use of training and education as a way of improving the delivery of preventive care services was mentioned by six of the ACOs interviewed. Most often this meant that ACO staff would provide training to practice staff in circumstances either where the practice was underperforming on a specific preventive care measure or when the ACO leadership wanted to promote a new preventive care initiative:

[The quality assistants will] bring that information [on underperformance against a specific target] back to the practice to meet with the practice manager and medical assistants just to show them that they are actually missing some gaps in care. They’ll also do remedial training if needed.

Another ACO told us about their annual “practice manager boot camp” where examples of best practice are shared between providers.

##### Use of Technology and the Electronic Health Record

Eight ACOs mentioned how technology either helps or hinders their delivery of preventive care services. Commonly ACOs reported using a central data warehouse to identify care gaps and then communicated this information to practices using the EHR.

Communication between different clinical providers, and between practices and patients, through either the EHR or an electronic patient portal was also used to identify and address preventive care needs. One physician described how their ACO was using the patient portal to find out if patients had received specific preventive health services:

we had a list of our patients who had open gaps, and were able to send emails, portal messages to these patients, asking them… we’d just like to get some dates on some of your preventive health and through that campaign…they would respond. It would automatically go into our electronic medical record and close the gap.

Conversely, an inadequately functioning EHR can also be a barrier to identifying which patients need specific preventive care services. For example, an ACO practice transformation coach told us how the EHR in some practices was not appropriately recording patients who were undertaking fecal immunochemical testing as an alternative to colonoscopy for colon cancer screening, meaning that patient screening records and practice quality scores were not accurate.

## DISCUSSION

In this study, we used quantitative and qualitative data to gain insights into how ACOs approach the delivery of preventive care services. Our interviews suggest important opportunities for improving care arising from the mechanisms used by ACOs and their motivations.

Our quantitative results indicate that Medicare ACOs who report that their providers are comprehensively engaged in patient care planning, including using reminders for preventive care, are also likely to rate themselves as having higher capabilities in various aspects of care management, performance measurement, and quality improvement. However, contrary to our expectations, reporting higher capabilities in these areas was not associated with achieving higher preventive quality scores or shared savings.

Our interview data, however, suggest that those working in ACOs that have achieved shared savings believe that similar mechanisms—care management programs, physician performance measurement, and closing care gaps (through reminders, for example)—are important for delivering preventive care services. The lack of association in the quantitative data could be explained by limitations of CMS quality measures and the challenges of achieving shared savings, or that additional mechanisms to those identified in the NSACO are also necessary to improve preventive quality scores and achieve shared savings. Our interview data provide possible examples of such mechanisms: such as increasing the uptake of AWVs (ACO uptake was just 30% in 2015, and AWVs can increase screening and vaccination rates^14, 26^) and implementing a more comprehensive system-wide approach to closing gaps in preventive care service use across the patient population (to specific patient groups, through care programs, and as part of routine care). Furthermore, it may be helpful for ACOs to identify whether their use of practice education and the EHR (as identified previously^13^) supports or hinders this work. ACO motivations for addressing patients’ preventive care needs identified in our interviews also provide insights for how CMS (and other payers) might motivate ACOs to change behavior—for example, through changing quality outcome measures or reimbursement rates.

Our results (except for the Medicare-specific AWV) are likely to be applicable to all payers and to patients with differing levels of complexity. Indeed, 13 of the 18 ACOs interviewed had ACO contracts outside of Medicare, and quality outcomes from other payers often include preventive care domains.^27^ And although incentives may differ, the mechanisms and facilitators that we identify may also be relevant to other managed care organizations.

Aside from closing preventive care gaps through care management and care coordination programs, interviews identified few mechanisms specifically addressing the preventive care needs of clinically complex patients despite this patient group being a focus of the interview. Instead, mechanisms were applicable across the entire ACO patient population. This may be because ACOs find that preventive care service uptake among patients with complex needs is already adequately addressed or because there is not a strong enough incentive for ACOs to develop more preventive care programs specifically targeting this patient group. Such programs are likely to be resource intensive, focusing on secondary rather than primary prevention (for example, behavioral interventions to reduce complications among patients with diabetes); as such, they may be less appealing to ACOs. Given that vulnerable, disabled, and minority patients (who are more likely to have complex clinical needs) use fewer preventive services and have more limited access to ACOs than less vulnerable patients, failing to target clinically complex patients may exacerbate inequalities in preventive care delivery.^14, 28–30^ Conversely, for some patients with complex needs—in particular those with limited life expectancy—providing certain preventive care services such as invasive screening may be clinically inappropriate.^31^

Among those interviewed, the motivations for ACOs to engage in preventive care activities were a combination of doing what is best for patients and meeting business priorities including risk adjustment scores and meeting quality targets. Therefore, payers should be aware that the choice of how to structure payments and what quality measures to use can have significant implications for ACO behavior. Additionally, more consideration should be given to non-financial motivators such as timely publication of performance data or using patient-reported outcomes.^32^

This study has limitations. Survey results are limited by sample size (although response rate was 64% with no evidence of response bias) and by potentially inaccurate responses because results are self-reported—the potential impact of this on results is not known. We were unable to fully adjust our survey results for patient case-mix, which may be confounding our quantitative results. Our interview sample included a heterogeneous group of ACOs in terms of geographic region, size, and mix of ACO contracts; however, findings may not be generalizable to all ACOs. For example, we do not know if the mechanisms to address preventive care needs identified through our interviews are lacking among ACOs that have not achieved shared savings—this would be of interest to explore in future research. Interviewees were asked about how they provide care to patients with complex needs rather than to all attributed patients; although our findings are largely applicable to all patients, we may not have been told about every activity or approach taken to providing preventive care services. We also do not know if mechanisms and motivations might differ between ACOs with different socio-economic or demographic patient profiles. And although interviewed ACOs described how they are addressing disease prevention, we do not have data on the effectiveness of these approaches—either in terms of their financial benefit or impact on quality scores. A useful follow-up study would be to explore the mechanisms and motivations identified in this study in more detail, in particular identifying possible barriers to their implementation.

This study identifies how some Medicare ACOs are addressing the preventive care needs of their patient population. Findings regarding the mechanisms used by ACOs and their motivations are relevant to other ACOs wanting to increase the delivery of preventive care services, and to payers wanting to influence ACO behavior.

## References

[1] U.S. Department of Health and Human Services. About the Affordable Care Act. https://www.hhs.gov/healthcare/about-the-aca/index.html. Published 2018. Accessed 15 August 2019.

[2] Seiler N, Malcarney M-B, Horton K, Dafflitto S (2014). Coverage of clinical preventive services under the Affordable Care Act: from law to access. Public Health Rep..

[3] Medicare Learning Network. Medicare Preventive Services. https://www.cms.gov/Medicare/Prevention/PrevntionGenInfo/medicare-preventive-services/MPS-QuickReferenceChart-1.html. Published 2018. Accessed 15 August 2019.

[4] RTI International. Accountable Care Organization 2017 Quality Measure Narrative Specifications. Waltham, MA; 2017. https://www.cms.gov/Medicare/Medicare-Fee-for-Service-Payment/sharedsavingsprogram/Downloads/2017-Reporting-Year-Narrative-Specifications.pdf.

[5] Borsky A, Zhan C, Miller T, Ngo-Metzger Q, Bierman AS, Meyers D (2018). Few Americans Receive All High-Priority, Appropriate Clinical Preventive Services. Health Aff..

[6] Han X, Robin Yabroff K, Guy GP, Zheng Z, Jemal A (2015). Has recommended preventive service use increased after elimination of cost-sharing as part of the Affordable Care Act in the United States. Prev Med (Baltim)..

[7] Jensen GA, Salloum RG, Hu J, Ferdows NB (2015). Tarraf W. A slow start: Use of preventive services among seniors following the Affordable Care Act’s enhancement of Medicare benefits in the U.S. Prev Med (Baltim)..

[8] Lau JS, Adams SH, Park MJ, Boscardin WJ, Irwin CE (2014). Improvement in preventive care of young adults after the affordable care act: the affordable care act is helping. JAMA Pediatr..

[9] Ganguli I, Souza J, McWilliams JM, Mehrotra A (2017). Trends in Use of the US Medicare Annual Wellness Visit, 2011-2014. JAMA..

[10] Chung S, Lesser LI, Lauderdale DS, Johns NE, Palaniappan LP, Luft HS (2015). Medicare Annual Preventive Care Visits: Use Increased Among Fee-For-Service Patients, But Many Do Not Participate. Health Aff..

[11] **Muhlestein D**, **Saunders R**, **Richards R**, **McClellan M**. Recent Progress In The Value Journey: Growth Of ACOs And Value-Based Payment Models In 2018. Health Affairs Blog. 10.1377/hblog20180810.481968. Published 2018.

[12] Maciosek MV, Coffield AB, Flottemesch TJ, Edwards NM, Solberg LI (2010). Greater Use Of Preventive Services In U.S. Health Care Could Save Lives At Little Or No Cost. Health Aff..

[13] Albright BB, Lewis VA, Ross JS, Colla CH (2016). Preventive Care Quality of Medicare Accountable Care Organizations: Associations of Organizational Characteristics With Performance. Med Care..

[14] Ganguli I, Souza J, McWilliams JM, Mehrotra A (2018). Practices Caring For The Underserved Are Less Likely To Adopt Medicare’s Annual Wellness Visit. Health Aff..

[15] Centers for Medicare and Medicaid Services. Physician fee schedule. https://www.cms.gov/apps/physician-fee-schedule/. Published 2018. Accessed 15 August 2019.

[16] Colla CH, Lewis VA, Shortell SM, Fisher ES (2014). First National Survey Of ACOs Finds That Physicians Are Playing Strong Leadership And Ownership Roles. Health Aff..

[17] Wu FM, Shortell SM, Lewis VA, Colla CH, Fisher ES (2016). Assessing Differences between Early and Later Adopters of Accountable Care Organizations Using Taxonomic Analysis. Health Serv Res..

[18] Comfort Leeann N., Shortell Stephen M., Rodriguez Hector P., Colla Carrie H. (2018). Medicare Accountable Care Organizations of Diverse Structures Achieve Comparable Quality and Cost Performance. Health Services Research.

[19] StataCorp. 2017. Stata Statistical Software: Release 15. College Station, TX: StataCorp LLC.

[20] Lewis VA, Colla CH, Schoenherr KE, Shortell SM, Fisher ES (2014). Innovation in the Safety Net: Integrating Community Health Centers Through Accountable Care. J Gen Intern Med..

[21] RTI International. Accountable Care Organization 2015 Program Analysis Quality Performance Standards Narrative Measure Specifications. Waltham, MA; 2015. https://www.cms.gov/medicare/medicare-fee-for-service-payment/sharedsavingsprogram/downloads/ry2015-narrativespecifications.pdf.

[22] NVivo qualitative data analysis software; QSR International Pty Ltd. Version 11, 2015.

[23] **Miles M**, **Huberman M**, **Saldaña J.** Qualitative Data Analysis: A Methods Sourcebook. 3rd Editio. Thousand Oaks: SAGE Publications, Inc; 2014.

[24] Medicare.gov. Yearly "Wellness" visits. https://www.medicare.gov/coverage/yearly-wellness-visits. Published 2019. Accessed 15 August 2019.

[25] Centers for Medicare and Medicaid Services. Shared Savings and Losses and Assignment Methodology Specifications Version 5. Baltimore, MD; 2017. https://www.cms.gov/Medicare/Medicare-Fee-for-Service-Payment/sharedsavingsprogram/Downloads/Shared-Savings-Losses-Assignment-Spec-V5.pdf. Accessed 15 August 2019.

[26] Camacho F, Yao NA, Anderson R (2017). The Effectiveness of Medicare Wellness Visits in Accessing Preventive Screening. J Prim Care Community Health..

[27] Kaufman Brystana G., Spivack B. Steven, Stearns Sally C., Song Paula H., O’Brien Emily C. (2017). Impact of Accountable Care Organizations on Utilization, Care, and Outcomes: A Systematic Review. Medical Care Research and Review.

[28] Yasaitis LC, Pajerowski W, Polsky D, Werner RM (2016). Physicians’ Participation In ACOs Is Lower In Places With Vulnerable Populations Than In More Affluent Communities. Health Aff (Millwood)..

[29] Lewis VA, Larson BK, McClurg AB, Boswell RG, Fisher ES (2012). The promise and peril of accountable care for vulnerable populations: a framework for overcoming obstacles. Health Aff (Millwood)..

[30] Colla CH, Lewis VA, Kao L-S, O’Malley AJ, Chang C-H, Fisher ES (2016). Association Between Medicare Accountable Care Organization Implementation and Spending Among Clinically Vulnerable Beneficiaries. JAMA Intern Med..

[31] Resnick MJ, Graves AJ, Thapa S (2018). Medicare Accountable Care Organization Enrollment and Appropriateness of Cancer Screening. JAMA Intern Med..

[32] Phipps-Taylor M, Shortell SM (2016). More Than Money: Motivating Physician Behavior Change in Accountable Care Organizations. Milbank Q..

